# Treatment with angiotensin II in COVID-19 patients may not be beneficial

**DOI:** 10.1186/s13054-020-03233-6

**Published:** 2020-09-04

**Authors:** Susanne Rysz, Francesca Campoccia Jalde, Anders Oldner, Lars I. Eriksson, Johan Lundberg, Malin Jonsson Fagerlund

**Affiliations:** 1 Department of Medicine Solna, Karolinska Institutet, Stockholm, Sweden; 2 Function Perioperative Medicine and Intensive Care, Karolinska University Hospital, Stockholm, Sweden; 3 Department of Physiology and Pharmacology, Karolinska Institutet, Stockholm, Sweden; 4 Department of Clinical Neuroscience, Karolinska Institutet, Stockholm, Sweden; 5 Department of Neuroradiology, Karolinska University Hospital, Stockholm, Sweden

Dear Editor,

We read with great interest the recent article by Zangrillo et al. regarding infusion of angiotensin II (ANGII) in COVID-19 [1], stating that ANGII vasopressor treatment may be logical in the setting of COVID-19 patients requiring vasopressor support. The authors refer to the ATHOSIII trial as support for the use of ANGII in catecholamine-resistant vasodilatory shock despite the known concern for thrombotic and infectious complications associated with ANGII [2]. In addition, the authors suggest to use ANGII in COVID-19 patients recently exposed to angiotensin-converting enzyme inhibitors. We believe that both these statements raise some concern.

SARS-CoV-1 downregulates ACE2 with a subsequent increase in ANGII levels creating a disruption akin to over activating the renin-angiotensin-aldosterone system (RAAS) [3]. In a recent COVID-19 case series, ANGII levels were markedly elevated and linearly associated with viral load and lung injury [4]. Moreover, in a prepublished report currently under journal review, infusion of ANGII in a porcine model rapidly (within hours) induced a clinical syndrome closely reflecting the one seen in COVID-19 patients, including histological changes in the lungs with severe thickening of the alveolar walls, possible hyaline membranes, and clotting of vessels, as previously reported in the human COVID-19 phenotype [5].

We suggest that much of the pathophysiology in ICU patients with COVID-19 is potentially driven by a loss of the inhibition of the RAAS, causing supranormal concentrations of ANGII [5]. In our opinion, the use of ANGII in COVID-19 patients is therefore at present most questionable. Rather, we propose further evaluation of a plausible contributing mechanism of RAAS behind pathophysiology seen in COVID-19.

## Data Availability

Not applicable.
