# Supplementary material for: Treatment with angiotensin II in COVID-19 patients may not be beneficial
Source: Crit Care. 2020 Sep 4;24:546. doi: 10.1186/s13054-020-03233-6 (PMC7472408; doi:10.1186/s13054-020-03233-6)
Supplement: Related texts

# Reply: Authors’ response

**Authors:** Alberto Zangrillo, Giovanni Landoni, Luigi Beretta, Federica Morselli, Ary Serpa Neto, Rinaldo Bellomo

We thank Dr. Rysz and colleagues for their correspondence [6].

In response, we note that their concerns about the increased risk of thrombosis and infection with angiotensin II have no statistical substance. In addition, for objectivity, other adverse effects would also have to be similarly considered, such as a 34% reduction in adverse effects leading to discontinuation compared to placebo, or the 34% reduction in respiratory adverse events, or the absolute 6.4% reduction in serious adverse events compared to placebo [7].

The endocytosis of ACE2 following contact with the COVID-19 virus could be decreased by conformational changes in ACE2 secondary to angiotensin II binding to it. In addition, such binding could itself decrease the ability of the virus to bind to the receptor and enter the cell. Such hypothetical interactions, however, remain unmeasured. Moreover, it is not a surprise that angiotensin II levels are higher in patients with greater viral load and illness severity. Such high levels can easily represent the response to inflammatory vasodilatation and the body’s attempt to restore perfusion pressure.

Finally, the infusion of angiotensin II in the porcine model cited by the correspondents is clearly irrelevant to angiotensin therapy in humans as described in our report [1]. We administered 20 ng/kg/min [1], while the investigators escalated to 80 ng/kg/min within 60 min and then all the way to 240 ng/kg/min. In other animals, the dose was up to 640 ng/kg/min. At such toxic doses which were 10 to 30 times those administered to our patients, it is no surprise that toxic, even lethal side effects developed.

Until more relevant experiments are performed or clear evidence emerges that angiotensin II infusion is injurious in COVID-19 patients, the data are strong that angiotensin II is a safe and effective vasopressor agent [8] especially in patients with high renin levels [9].
